# Development of gold nanoparticles stabilized by PLGA and PVA for application in photothermal therapy

**DOI:** 10.3389/fcell.2026.1759401

**Published:** 2026-02-05

**Authors:** Soraia Borges, Natanael Fernandes, André F. Moreira

**Affiliations:** 1 RISE-Health, Departamento de Ciências Médicas, Faculdade de Ciências da Saúde, Universidade da Beira Interior, Covilhã, Portugal; 2 AEROG-LAETA, Aerospace Sciences Department, Universidade da Beira Interior, Covilhã, Portugal; 3 BRIDGES - Biotechnology Research, Innovation, and Design of Health Products, Polytechnic of Guarda, Guarda, Portugal

**Keywords:** gold nanoparticles, hyperthermia, nanoclusters, photothermal therapy, PLGA

## Abstract

**Introduction:**

In today's world, cancer remains a major public health problem. Consequently, there has been a focused investigation of alternative therapeutic approaches to address this ongoing health concern. In this study, PLGA-gold nanomaterials were produced through various processes to determine whether these variations affected their structural integrity and, consequently, their physicochemical properties.

**Methods:**

The PLGA-gold nanomaterials were produced through an oil-in-water (O/W) process (Formulation A) or a water-in-oil-in-water (W1/O/W2) process (Formulation B). The nanomaterials' physiscochemical properties were characterized by electron microscopy, dynamic light scattering, and UV-vis. Photothermal studies were performed using a 808 nm NIR laser and the PLGA-gold nanomaterials cytocompatibility was evaluated using resazurin.

**Results:**

Both methods originated particles with similar size and charge, 276.8 and –19.6 mV for formulation A and 317.5 nm and –18.6 mV for formulation B. Nevertheless, the TEM images revealed structural differences, with formulation A presenting the gold spheres clustered in the particle nucleus, whereas in formulation B the gold spheres were found in the outer PLGA shell. Moreover, upon irradiation with a NIR laser (808 nm, 1.7 W cm–2, 10 min), the particles showed a concentration dependent photothermal effect, promoting a temperature increase of 21 °C and 13.9 °C for formulation A and B at 400 μg/mL, respectively. Additionally, preliminary cellular assays demonstrated that the PLGA-gold nanoparticles are cytocompatible, with both FibH and HeLa cells exhibiting a cellular viability of approximately 100%.

**Conclusion:**

Thus, these results underline the potential the PLGA-gold nanoparticles, particularly formulation A, for advanced applications in nanomedicine. In the future the encapsulation of drugs will be evaluated in order to characterise both the uptake and the cytotoxic capacity of this multifunctional nanomaterial. Additionally, the utilization of more complex in vitro models, such as tumor spheroids and animal models, will then be essential to determine the therapeutic potential of PLGA/gold nanoparticles.

## 1 Introduction

Cancer continues to pose a major global health challenge, despite considerable advances in detection and treatment in recent years (Kiri and Ryba, 2024; Bray et al., 2024). According to the World Health Organization, cancer is second leading cause of death worldwide, underscoring the urgent need for innovative therapeutic strategies (Bray et al., 2024). In response to this pressing challenge, research has increasingly focused on the development of nanoparticle-based therapies, such as hyperthermia induced by electromagnetic fields, radio frequencies, ultrasounds, and light (Ghaffarlou et al., 2024; Fernandes et al., 2020; Rodrigues et al., 2019a). These approaches represent potential alternatives to conventional anticancer treatments, offering more targeted and effective options. Particularly, light-activated hyperthermia mediated by nanoparticles has emerged as a highly promising cancer treatment strategy due to its non-invasive nature, high precision in tumor targeting, and minimal damage to surrounding healthy tissues (Fernandes et al., 2020; Salimi et al., 2022).

Metallic nanoparticles, such as silver nanoparticles (AgNPs) and gold nanoparticles (AuNPs), have been extensively investigated for use in cancer photothermal therapy (PTT) (Fernandes et al., 2020; Gonçalves et al., 2022; Alamdari et al., 2022; Kadkhoda et al., 2022). Among these, AuNPs are known for their low toxicity and nonimmunogenic nature. Additionally, AuNPs' surface plasmon resonance (SPR) endows them with unique optical properties, including the ability to absorb light and convert it into heat (Moreira et al., 2025; Gupta and Malviya, 2021). Laser light in the near-infrared (NIR) region is typically used to activate AuNPs, as it exhibits reduced interaction with biological tissues, allowing for deeper tissue penetration and minimal damage to surrounding healthy cells (Hemmer et al., 2016). In this context, the SPR absorption peak of AuNPs can be tuned to the NIR region by optimizing various physicochemical parameters, such as the size, shape, and structural organization (Gonçalves, 2020). Accordingly, a variety of AuNPs morphologies have been engineered, such as nanospheres, nanorods, nanostars, and nanocages as gold cores (Fernandes et al., 2021; Yang et al., 2021; D et al., 2025; Pal et al., 2022). Moreover, the surface functionalization of AuNPs plays also a crucial role in modulating their photothermal performance and biological interactions, influencing factors such as cancer cells, cellular uptake, biodistribution, and toxicity (Rodrigues et al., 2019a; Nicol et al., 2015).

Gold nanospheres represent the most common and structurally stable shape form of AuNPs. Nevertheless, these nanomaterials primarily absorb light in the visible range, limiting their effectiveness for NIR-triggered PTT (Demers et al., 2017). Nonetheless, organizing gold nanospheres into clusters or shell-like assemblies can induce a redshift of the SPR absorption peak, thereby enhancing their applicability in NIR-triggered PTT (Iodice et al., 2016; Fernandes et al., 2025). Specifically, the clustering of gold nanospheres can be achieved through the encapsulation within polymer matrices, capsules, or lipidic-based vesicles (Iodice et al., 2016; Jang et al., 2022; Moreira et al., 2017; Tatykhanova et al., 2025). In particular, polymers have been shown to function as stabilizing, structuring, and reducing agents, playing a key role in the synthesis of gold nanoparticles with various structures and organizational arrangements (Tatykhanova et al., 2025; Das et al., 2023; Tabesh et al., 2024; Vazirieh Lenjani et al., 2022; Yao et al., 2023).

Poly (D, L-lactic-co-glycolic acid) (PLGA) is a biodegradable and biocompatible copolymer composed of two monomers: D, L-lactic acid and glycolic acid, which are linked together to form a chain-like structure (Tonbul and Çapan, 2023; Swider et al., 2018). The versatility and promising properties of PLGA have led to the approval of several PLGA-based drug delivery systems and medical devices by the Food and Drug Administration (FDA) and the European Medicines Agency for clinical application (Danhier et al., 2012). Among its various applications, PLGA can be employed to produce nanoparticles and nanocapsules through water-in-oil emulsification methods, followed by the organic solvent removal via evaporation (Soppimath et al., 2001). Several studies have demonstrated the use of PLGA nanoparticles for gene and drug delivery, highlighting their ability to provide a controlled and sustained drug release (Gaspar et al., 2015; López-Royo et al., 2021; Wang et al., 2010). Moreover, PLGA nanoparticles have shown the capacity to cross biological barriers, such as the blood–brain barrier, and to be efficiently internalized by cells, underscoring their potential as a versatile platform for advanced therapeutic strategies (Zhi et al., 2021; Chang et al., 2012; Zhang et al., 2021). PVA is a widely used, non-toxic, and water-soluble polymer that enhances colloidal stability and reduces nonspecific protein adsorption, mitigating undesired cellular interactions (Barrett et al., 2001).

The present study aimed to explore the development of gold nanoparticles stabilized by PLGA and PVA for application in photothermal therapy. Our approach was based on previous experience in creating PLGA microcapsules for transporting and delivering drugs and chemotherapeutics (Moreira et al., 2017; Gaspar et al., 2015). For that purpose, two distinct emulsion-based fabrication methods were evaluated to mediate the clustering and stabilization of gold nanospheres. We hypothesized that by using an oil-in-water (O/W) or water-in-oil–in-water (W/O/W) methodology we could modulate the gold nanospheres accumulation in the core or polymeric capsule of the resulting nanoparticles. Additionally, the two different methodologies can also impact on the AuNPs entrapment efficiency, as well as on the nanomaterials’ physicochemical properties and photothermal performance. Therefore, the development of PLGA and PVA stabilized gold nanoparticles can result novel therapeutics with favorable biological interactions as well as photothermal and bioimaging potential that can contribute to a novel generation of antitumoral therapies.

## 2 Materials and methods

### 2.1 Materials

Hydrogen tetrachloroaurate (III) hydrate (HAuCl_4_) was purchased from Alfa Aesar (Karlsruhe, Germany). Dulbecco’s Modified Eagle medium-high glucose (DMEM-HG), Dulbecco’s Modified Eagle Medium/Nutrient Mixture F-12 (DMEM-F12), phosphate-buffered saline solution, ethanol (EtOH), trisodium citrate (NaCt), trypsin, resazurin were purchased from Sigma-Aldrich (Sintra, Portugal). Human negroid cervix epithelioidcarcinoma (HeLa cells) (ATCCs CCL-2TM) were acquired from ATCC (Middlesex, United Kingdom). Primary normal human dermal fibroblast (FibH) cells were bought from Promocell (Heidelberg, Germany). Cell culture t-flasks were obtained from Orange Scientific (Braine-l’Alleud, Belgium). Double deionized and filtered water (ultrapure water) was obtained by using a Milli-Q Advantage A10 Ultrapure Water Purification System (0.22 μm filtered; 18.2 MΩ/cm at 25 °C). Poly (D, L-lactic-co-glycolic acid) (PLGA, 75:25, M_W_: 76,000 g/mol) and Polyvinyl Alcohol (PVA; M_W_: 31,000 g/mol) were obtained from Sigma-Aldrich (Sintra, Portugal). Tri-sodium citrate anhydrous was acquired from MERK (Darmstadt, Germany).

### 2.2 Methods

#### 2.2.1 Synthesis of nanospheres

Gold nanospheres were synthesised by adapting a previously described method (Dong et al., 2020). In this reaction, 250 µL of HAuCl_4_ (0.05 M) were added to 49.75 mL of ultrapure water (resistivity 18.2 mΩ/cm) under vigorous stirring at 100 °C. After 15 min, 1.053 mL of NaCt aqueous solution (10 mg/mL ultrapure water) was added to the solution and left to react for 10 min. The resulting gold nanospheres were then recovered and stored at 4 °C until use.

#### 2.2.2 Synthesis of PLGA–Gold nanoparticles

PLGA-Gold nanoparticles were prepared by carrying out an Emulsification-Evaporation method (Moreira et al., 2017; Danhier et al., 2012). For formulation A, 5 mL of the pre-prepared gold nanospheres were added to the water phase, which consisted of 0.25 mL of PVA (40 mg/mL). The mixture was stirred for 15 min. Following this, an oil phase containing 2 mL of PLGA (5 mg/mL) was added. The solution was then emulsified through sonication in a cold bath for a period of 30 min. Thereafter, a further 6 mL of PVA (20 mg/mL) was added to the primary emulsion and sonicated for a period of 30 min, producing an Oil-in-water (O/W1) emulsion. In order to assess the effect of these alterations on the resultant emulsion, a second formulation (B) was prepared and subjected to the same process as formulation A. To this end, 3 mL of the gold nanospheres, were added to the water phase, which comprised 0.25 mL of PVA (40 mg/mL). The mixture was stirred for 15 min. Thereafter, an oil phase containing 6.5 mL of PLGA (1.5 mg/mL) was added, after which the solution was emulsified through sonication in a cold bath for 30 min to obtain the primary water-in-oil (W1/O) emulsion. Subsequently, an additional 6 mL of PVA (20 mg/mL) was introduced into the primary emulsion and sonicated for a duration of 30 min, thereby yielding the secondary emulsion, water-in-oil-in-water (W1/O/W2).

The resulting nanoparticles were recovered by centrifuging (20 min, at 10,000g and 25 °C) and resuspended in water.

### 2.3 Characterization of nanocarriers’ physicochemical properties

#### 2.3.1 Morphological characterization

The structural and morphological properties of the PLGA-Gold nanoparticles were studied in detail using transmission electron microscopy (TEM−Hitachi−HT7700, Tokyo, Japan) at an accelerating voltage of 80 kV. For that purpose, a drop of the sample was placed on the TEM grid and allowed to dry at room temperature before analysis in the TEM microscope. Additionally, the Scanning Electron Microscopy (SEM) was used to analyse the surface and morphology of the PLGA-Gold nanoparticles. The samples were placed on a circular glass coverslip, dried at room temperature, and coated with gold using a Quorum Q150R ES sputter coater (Quorum Technologies, Ltd., Laughton, East Sussex, UK). Then, the circular glass coverslip was mounted on aluminium stubs using araldite tape. The SEM images, at different magnifications, were then obtained using a Hitachi S-3400 N Scanning Electron Microscope (Hitachi, Tokyo, Japan) operated at 20 kV.

#### 2.3.2 Size and zeta potential analysis

The hydrodynamic mean size, size distribution, and zeta potential of the PLGA-Gold nanoparticles were determined using a Zetasizer Nano ZS instrument (Malvern Instruments, Worcestershire, UK) at 25 °C. For all measurements, the nanoparticles were resuspended in ultrapure water, and the data was collected in a disposable capillary cell.

#### 2.3.3 Ultraviolet-visible spectroscopy analysis

The ultraviolet–visible (UV-vis) spectra of the nanoparticles were recorded at a scanning rate of 300 nm per minute, 1 nm step, and wavelength range of 300–1000 nm using a UV-vis spectrophotometer (Thermo Scientific Evolution™ 201 Bio UV-vis Spectrophotometer, Thermo Fisher Scientific Inc., USA), to evaluate the success of the nanoparticles synthesis.

#### 2.3.4 *In vitro* photothermal measurements

The evaluation of the PLGA-Gold nanoparticles’ *in vitro* photothermal capacity was performed as previously described in the literature (Rodrigues et al., 2019b). Briefly, nanoparticles at a concentration of 200 μg/mL and 400 μg/mL were irradiated with a NIR laser (808 nm, 1.7 W cm^-2^ for 10 min). Then, the variation of the temperature was measured at different time points (from 1 to 10 min) using a thermocouple sensor with an accuracy of 0.1 °C.

Photothermal conversion efficiency was calculated using the following Equation 1 (Fernandes et al., 2025):

$$\eta =\frac{h\;x\;S\;\left(Tmax-Tamb\right)-Qdis}{I\;\left(1-{\;10}^{-A808}\right)}$$ (1)

$$\text{hS}=\frac{m\times C}{\tau_{s}}$$ (2)

T_max_ refers to the peak temperature reached during laser irradiation, while T_amb_ corresponds to the room temperature. Q_dis_ accounts for the heat dissipated due to absorption by the surrounding medium and container. I represent the NIR laser power density (1.7 W/cm^2^), and A808 corresponds to the particles’ optical absorbance at 808 nm. The term hS was obtained from Equation 2, where S is the surface area of the container and h the heat transfer coefficient. C is the specific heat capacity of water (4.2 J/g°C), m is the mass of water used (0.2 g), and τ s is the time constant of the thermal system, calculated following Equation 3:

$$\tau s=-\frac{t}{\ln\left(Ɵ\right)}$$ (3)

$$Ɵ=\frac{T-T\;amb}{T\;\max-T\;amb}$$ (4)

t is the irradiation duration (600 s) and θ is a dimensionless temperature parameter derived from Equation 4. The ambient temperature was set at ∼20 °C throughout the experiments.

### 2.4 *In vitro* cell studies

The cytocompatibility of the PLGA/Gold nanoparticles was evaluated using a resazurin-based assay. For this assay, HeLa or FibH cells were seeded in 96-well flat-bottom culture plates at a density of 10,000 cells per well and cultured with 200 μL of culture medium (DMEM-HG and DMEM-F12, respectively) for 24 h at 37 °C in a humidified atmosphere containing 5% CO2. Then, the media was removed, and the cells were incubated with different concentrations (50–400 μg/mL) of the PLGA-Gold nanoparticles formulations. At 24, 48, and 72 h of incubation, the medium was replaced by 110 μL of 10% (v/v) resazurin solution and incubated for 4 h. The resulting resorufin fluorescence was then quantified using a spectrofluorometer (Spectramax Gemini XS, Molecular Devices LLC, USA) at an excitation/emission wavelength of λ_ex_ = 560 nm and λ_em_ = 590 nm. Cells incubated with absolute EtOH were defined as the positive control (K^+^), whereas cells cultured without nanoparticles’ exposure were used as the negative control (K^−^).

### 2.5 Statistical analysis

Statistical analysis of the obtained results was performed using GraphPad Prism v.8.0 software (Trial version, GraphPad Software, CA, USA). Data are presented as the mean ± standard deviation (s.d.). One-way analysis of variance (ANOVA) with the Student–Newman–Keuls post-test was used to compare different groups. A value of p < 0.05 was considered statistically significant.

## 3 Results and discussion

### 3.1 Characterization of the PLGA/Gold nanoparticles

PLGA/Gold nanoparticles were produced through an O/W (formulation A) or W/O/W (formulation B) emulsion methods to study the impact in their structural integrity and physicochemical properties. During the emulsification, gold nanospheres (19 nm in size) were added to the water phase and the nanoparticles formation was finalized by evaporating the organic solvent (i.e., dichloromethane) under magnetic agitation.

**FIGURE 1:**
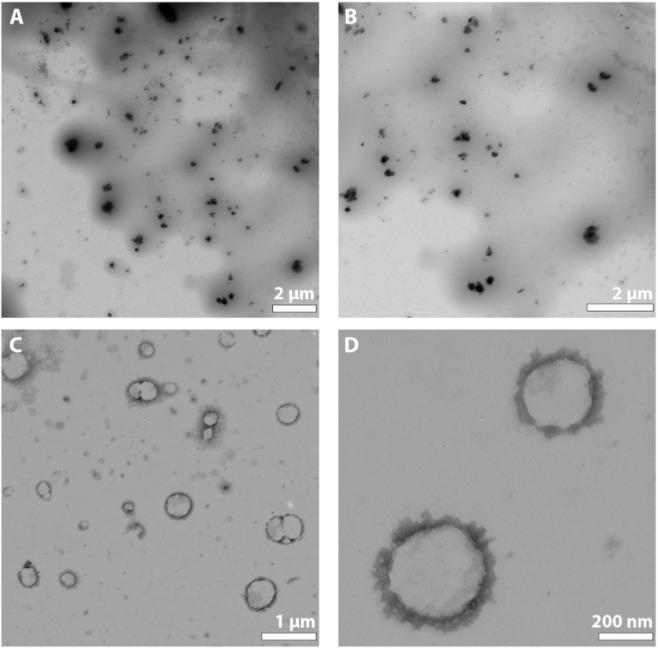
TEM images of formulation A **(A,B)** and formulation B **(C,D)**.

The analysis of transmission electron microscopy (TEM) images indicates significant disparities between both formulations (Figure 1). It is evident that Formulation A, produced via O/W emulsion, features gold nanoparticles within the capsule’s core. In contrast, Formulation B, prepared using a water-in-oil (W/O/W) approach, exhibits gold nanostructures encircling the core, embedded within the polymeric matrix resembling a nanocapsule. Nevertheless, both methodologies indicate the successful formation of the PLGA/Gold nanoparticles. During the emulsion process, PVA acts as a surfactant in the interface between the water and oil phases, stabilizing the PLGA and minimizing the oil coalescence (Gaspar et al., 2015).

**FIGURE 2:**
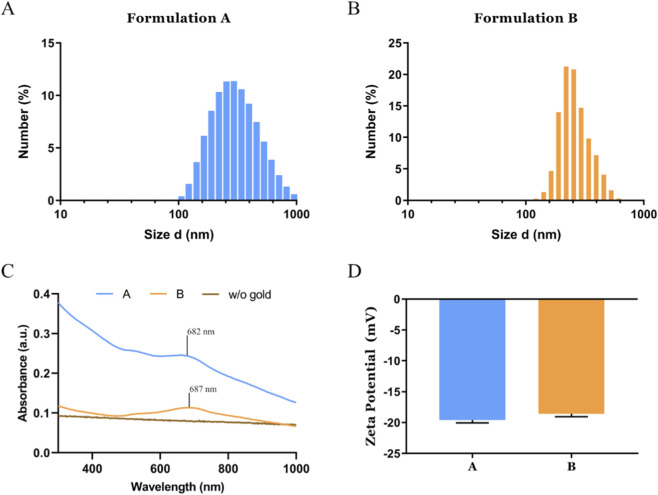
Physicochemical characterization of the PLGA/Gold nanoparticles. Size distribution analysis of formulation A **(A)** and formulation B **(B)**. UV-vis absorption spectra of the PLGA/Gold nanoparticles **(C)**. Analysis of the PLGA/Gold nanoparticles’ zeta potential, data presented as mean ± s.d., n = 3 **(D)**.

In consideration of these findings, UV-Vis-NIR spectra were also obtained to ascertain whether the location of gold nanoparticles within the capsule had any impact on the absorption spectra and, by extension, the photothermal potential. The absorption spectra of gold nanospheres show the characteristic absorption peak at 535 nm (Supplementary Figure S1). Nevertheless, no peak in this region can be identified in the A and B formulations (Figure 2). The results obtained demonstrated an enhancement in the red region of the spectra of the PLGA nanoparticles with the incorporation of gold nanospheres. This finding is consistent with the coupling of the plasmonic resonance of different gold nanospheres in proximity, i.e., entrapped in the nanomaterials (Yang et al., 2023; Husni et al., 2023; Zhuo, 2022). The augmented absorption in the red region of the spectra, including the NIR region, is pivotal for the utilisation of PLGA/gold nanoparticles in photothermal therapy, due to the selective activation of the nanomaterials with minimised off-target interactions, specifically with major biological constituents such as blood, proteins, and water (Hossain et al., 2022; Zhang et al., 2025).

The differences between the formulations were also assessed by measuring the mean size and particle distribution using the Dynamic Light Scattering (DLS). The resulting data demonstrate that formulations A and B have a mean size of 276,8 and 317,5nm, respectively. Moreover, the measured PDI values, 0.197 and 0.340, indicate that the PLGA/Gold nanoparticles have a homogeneous distribution.

In addition, the analysis of the zeta potential revealed that the production methodology exerts minimal influence on the surface charge of PLGA/gold nanoparticles. The formulation A and B exhibited surface charges of −19.6 and −18.6 mV, respectively. The negative surface charge of the PLGA/gold nanoparticles is consistent with the anionic nature of PVA and PLGA (Kaur et al., 2021; Lee et al., 2024; Robin et al., 2021). Furthermore, the surface charge values obtained for PLGA/Gold nanoparticles are close to the ideal values for biological applications, *i.e.*, ±10 mV, and can contribute to an extended blood circulation time (Ernsting et al., 2013; Li and Huang, 2008).

**FIGURE 3:**
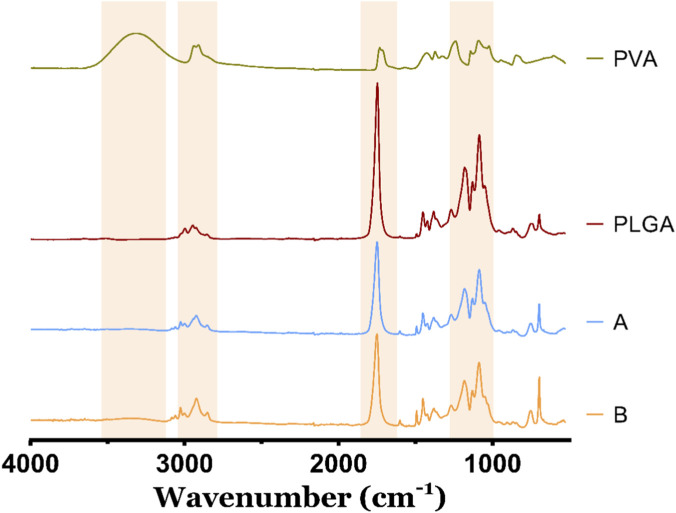
FTIR spectra of PVA, PLGA, and PLGA/Gold formulations A and B.

Besides the morphological and optical characterization, the composition of the PLGA/Gold nanoparticles was assessed through FTIR spectroscopy (Figure 3). The PLGA spectra present four characteristic bands, namely, at 2999–2952 cm^-1^ corresponding to the C-H stretching vibration, 1451–1381 cm^-1^ corresponding to C-H bending, and two stretching peaks, at 1747 cm^-1^ corresponding to the ester functional groups of the C=O stretching, and at 1085 cm^-1^ corresponding to C-O vibration (Wang et al., 2019; Chen et al., 2018). In turn, the PVA has characteristic bands at: 3316 cm^-1^, which is attributed to the O-H stretching vibration from the intermolecular and intramolecular hydrogen bonds; 2910 cm^-1^ that refers to the C–H stretching from alkyl groups; 1731 cm^-1^ due to the C=O stretching; and at 1100 cm^-1^ related with the C-O stretching (Mansur et al., 2008; Reis et al., 2006). The analysis of the spectra obtained for the A and B formulations reveals the presence of characteristic peaks and bands associated with PLGA and PVA. Specifically, the C–H stretching at 2910 cm^-1^ in PVA, the C=O stretching at 1747 cm^-1^, and the C-O vibration at 1100 cm^-1^ are noteworthy (Mir et al., 2016; Ramesan et al., 2024).

### 3.2 Evaluation of the photothermal potential

The UV-Vis-NIR results showed an enhancement in the red region of the spectrum for both formulations, thereby suggesting that both A and B formulations have the capacity to absorb and convert light into heat. This evaluation was conducted by irradiating the nanoparticles for 10 min under NIR laser, thereby providing a quantitative analysis of their photothermal conversion capabilities.

**FIGURE 4:**
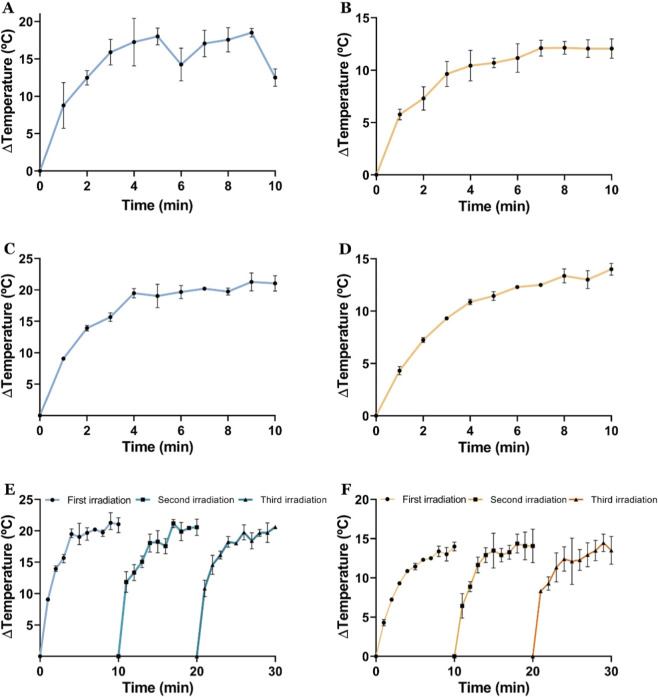
Characterization of the PTT capacity of PLGA/Gold nanoparticles. Temperature variation curves of aqueous solutions containing PLGA/Gold nanoparticles at a concentration of 200 μg/mL for formulations A **(A)** and B **(B)**, under NIR laser irradiation (808 nm, 1.7 W cm^-2^). Temperature response of PLGA/Gold nanoparticles at 400 μg/mL under NIR laser irradiation (808 nm, 1.7 W cm^-2^) for 10 min, for formulation A **(C)** and formulation B **(D)**. Thermal stability and reproducibility of the photothermal effect over three NIR cycles (808 nm, 1.7 W cm^-2^, 10 min) for formulation A **(E)** and formulation B **(F)**. Data presented as mean ± s.d., n = 3.

The obtained results show that the PLGA/Gold nanoparticles could mediate a constant increase in the media temperature during the 10 min of irradiation (Figure 4). Particularly, formulation A (200 μg/mL), after one irradiation, raised 12.5 °C the medium temperature, which reached the 21 °C increase, at the concentration of 400 μg/mL. Otherwise, the formulation B showed a temperature increase of 12 °C at 200 μg/mL and 13.9 °C at 400 μg/mL. Moreover, subsequent analyses demonstrated that formulation A exhibits a photothermal conversion efficiency of approximately 71%, whereas formulation B displays an efficiency of 53.5%. These photothermal results, particularly for formulation A, are in accordance with the photothermal capacity shown by other nanomaterials based on gold and PLGA (Hao et al., 2015; Xi et al., 2018). For example, Hao obtained a temperature increase of 11.8 °C and 20.2 °C for PLGA nanoparticles with a gold nanoshell at concentrations of 100 and 500 μg/mL, respectively. In addition, the photothermal capacity of the PLGA/gold nanoparticles was also assessed under multiple NIR laser irradiation cycles. As demonstrated in the results obtained, it can be observed that the heating profile remained constant during the three irradiation cycles.

**FIGURE 5:**
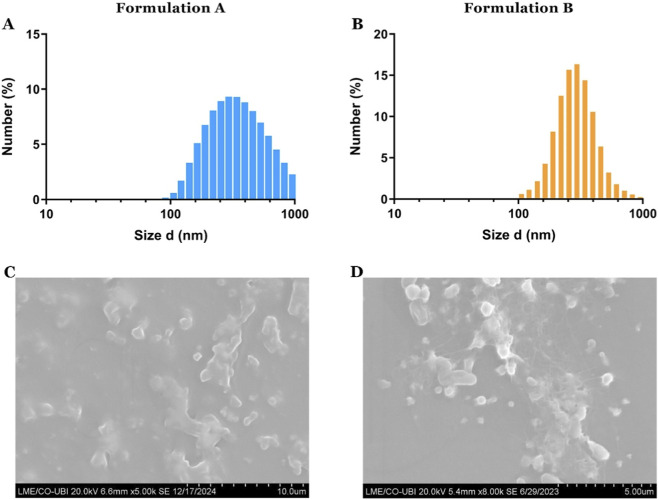
DLS analysis of formulation A **(A)** and formulation B **(B)**, after being subjected to NIR laser irradiation (808 nm, 1.7 W cm^-2^) for 10 min. SEM images of formulation A **(C)** and formulation B **(D)**, after NIR laser irradiation (808 nm, 1.7 W cm^-2^) for 10 min, showing the presence of film-like structures and degraded particles.

The findings indicate that both formulations are capable of converting light into heat, and the obtained temperature variations are within therapeutic values, mainly to sensitize the cancer cells to the action of other therapeutics (Fernandes et al., 2020; Xi et al., 2018). Subsequently, the PLGA/gold nanoparticles’ size was assessed after the irradiation with the NIR laser, showing a slight overall increase in the nanoparticles’ diameter (Figures 5A,B). This is indicative of the temperature-mediated degradation of the nanoparticles. Additionally, SEM analysis of the PLGA/gold nanoparticles (Supplementary Figure S2) and post-irradiation (Figures 5C,D) revealed an apparent structural damage to the polymeric capsule, along with the appearance of polymer-like films or degraded particles, which can be attributed to the polymers’ melting in response to the heat generated.

### 3.3 *In vitro* cell studies

#### 3.3.1 Biocompatibility of PLGA-Gold nanoparticles

**FIGURE 6:**
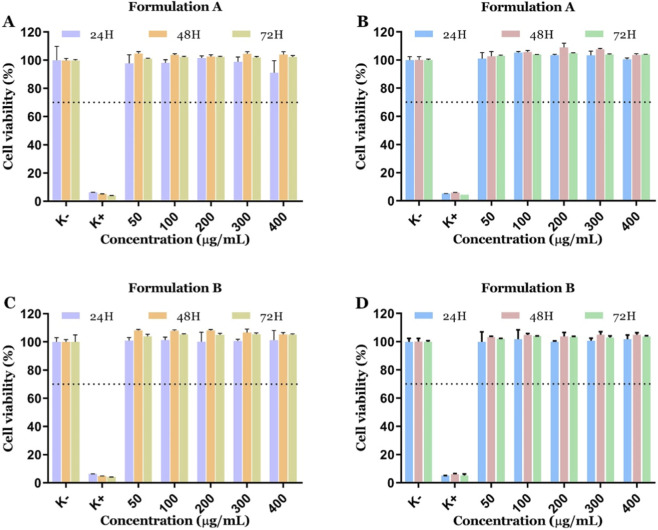
Cytocompatibility evaluation of PLGA/Gold nanoparticles on HeLa **(A,C)** and FibH cells **(B,D)** at 24, 48, and 72 h. Data are presented as mean ± s.d., n = 5.

The biocompatibility of the PLGA-Gold nanoparticles was evaluated both on FibH and HeLa cells. Therefore, the A and B formulations of PLGA-Gold nanoparticles were incubated for 24, 48, and 72 h, at concentrations ranging from 50 to 400 μg/mL, and the cellular viability was measured using the resazurin assay (Figure 6). According to the ISO 10993–5 “Biological evaluation of medical devices -Part 5: Tests for *in vitro* cytotoxicity”, a material has a cytotoxic effect when the cell viability is reduced by more than 30%. The obtained results show that both FibH and HeLa cells maintained the cell viability within the range considered biocompatible after 24, 48, and 72 h of incubation, even at the highest tested nanoparticle concentration (400 μg/mL). The minor, non-significant variations in cell viability observed, especially at increasing nanoparticle concentrations, may reflect subtle metabolic alterations and then be potentially indicative of low-level cellular stress. Nevertheless, these data are in accordance with the biocompatibility profile of PVA and PLGA polymers, which have FDA approval for applications in food packaging, biomedicine, and pharmaceutics (Danhier et al., 2012; Bobo et al., 2016; Sharma, 2016). PLGA undergoes hydrolysis into lactic and glycolic acids, which are naturally metabolized through physiological pathways, thereby limiting long-term tissue accumulation (Silva, 2015). PVA reduces nonspecific protein adsorption, mitigating undesired cellular interactions (Barrett et al., 2001). The combined PLGA/PVA stabilization forms a protective interface surrounding the gold core, reducing direct metal–cell contact. Additionally, the gold nanomaterials are also classified as biocompatible and the least toxic metal-based nanoparticles (Kang et al., 2020; Schrand et al., 2010). Collectively, these characteristics explain the overall biocompatibility of PLGA- and PVA-stabilized gold nanomaterials.

## 4 Conclusion

In this study, PLGA-gold nanoparticles were synthesised through two distinct processes. Two formulations were evaluated, formulation A (oil-in-water (O/W)) and formulation B (water-in-oil-in-water (W1/O/W2)). This study aimed to investigate the extent to which these processes might influence the structural integrity of the resulting nanoparticles, and consequently their physicochemical properties. Considering the data presented, it can be discerned that the two processes led to comparable outcomes in terms of size and charge, with formulation A presenting a superior photothermal capacity, reaching the maximum 21 °C increase at concentration of 400 μg/mL. The cell studies showed the excellent cytocompatibility of the PLGA-gold nanoparticles in both HeLa and FibH cells even at the maximum tested concentration (400 μg/mL) for both formulations. In summary this work showed that despite the different processes used during the production of PLGA-gold nanoparticles did not result in significant variations in the obtained outcomes. The major difference was related with the location of the gold nanospheres on the capsule, showed by the TEM images. In the case of formulation A, the nanospheres were positioned at the core of the capsule, whereas in sample B, they were situated within the capsule’s matrix. Furthermore, in the future the encapsulation of drugs will be evaluated in order to characterise both the uptake and the cytotoxic capacity of this multifunctional nanomaterial. Additionally, the utilization of more complex *in vitro* models, such as tumor spheroids and animal models, will then be essential to determine the therapeutic potential of PLGA/gold nanoparticles.

## Data Availability

The raw data supporting the conclusions of this article will be made available by the authors, without undue reservation.

## References

[B1] Alamdari S. G. Amini M. Jalilzadeh N. Baradaran B. Mohammadzadeh R. Mokhtarzadeh A. (2022). Recent advances in nanoparticle-based photothermal therapy for breast cancer. J. Control. Release 349, 269–303. 10.1016/j.jconrel.2022.06.050 35787915

[B2] Barrett D. Hartshome M. S. Hussain M. A. Shaw P. N. Davies M. C. (2001). Resistance to nonspecific protein adsorption by poly (vinyl alcohol) thin films adsorbed to a poly (styrene) support matrix studied using surface plasmon resonance. Anal. Chemistry 73 (21), 5232–5239. 10.1021/ac010368u 11721924

[B3] Bobo D. Robinson K. J. Islam J. Thurecht K. J. Corrie S. R. (2016). Nanoparticle-based medicines: a review of FDA-Approved materials and clinical trials to date. Pharm. Research 33 (10), 2373–2387. 10.1007/s11095-016-1958-5 27299311

[B4] Bray F. Laversanne M. Sung H. Ferlay J. Siegel R. L. Soerjomataram I. (2024). Global cancer statistics 2022: GLOBOCAN estimates of incidence and mortality worldwide for 36 cancers in 185 countries. CA A Cancer Journal Clinicians 74 (3), 229–263. 10.3322/caac.21834 38572751

[B5] Chang J. Paillard A. Passirani C. Morille M. Benoit J. P. Betbeder D. (2012). Transferrin adsorption onto PLGA nanoparticles governs their interaction with biological systems from blood circulation to brain cancer cells. Pharm. Research 29 (6), 1495–1505. 10.1007/s11095-011-0624-1 22167349

[B6] Chen L. Mei L. Feng D. Huang D. Tong X. Pan X. (2018). Anhydrous reverse micelle lecithin nanoparticles/PLGA composite microspheres for long-term protein delivery with reduced initial burst. Colloids Surfaces B Biointerfaces 163, 146–154. 10.1016/j.colsurfb.2017.12.040 29291500

[B7] Delgado-Corrales B. J. Chopra V. Chauhan G. (2025). Gold nanostars and nanourchins for enhanced photothermal therapy, bioimaging, and theranostics. J. Mater. Chem. B 13 (2), 399–428. 10.1039/d4tb01420k 39575861

[B8] Danhier F. Ansorena E. Silva J. M. Coco R. Le Breton A. Préat V. (2012). PLGA-based nanoparticles: an overview of biomedical applications. J. Controlled Release 161 (2), 505–522. 10.1016/j.jconrel.2012.01.043 22353619

[B9] Das T. Das S. Das D. (2023). Fabrication of core–shell beads, hollow capsules, and AuNP-embedded catalytic beads from an ultrasmall peptide hydrogel. Chem. Eng. J. 477, 147105. 10.1016/j.cej.2023.147105

[B10] Demers S. M. Hsieh L. J. H. Shirazinejad C. R. Garcia J. L. A. Matthews J. R. Hafner J. H. (2017). Ultraviolet analysis of gold nanorod and nanosphere solutions. J. Phys. Chem. C 121 (9), 5201–5207. 10.1021/acs.jpcc.6b09066

[B11] Dong J. Carpinone P. L. Pyrgiotakis G. Demokritou P. Moudgil B. M. (2020). Synthesis of precision gold nanoparticles using Turkevich method. KONA Powder Part. J. 37, 224–232. 10.14356/kona.2020011 32153313 PMC7062369

[B12] Ernsting M. J. Murakami M. Roy A. Li S. D. (2013). Factors controlling the pharmacokinetics, biodistribution and intratumoral penetration of nanoparticles. J. Controlled Release 172 (3), 782–794. 10.1016/j.jconrel.2013.09.013 24075927 PMC3891171

[B13] Fernandes N. Rodrigues C. F. Moreira A. F. Correia I. J. (2020). Overview of the application of inorganic nanomaterials in cancer photothermal therapy. Biomaterials Science 8 (11), 2990–3020. 10.1039/d0bm00222d 32355937

[B14] Fernandes N. Rodrigues C. F. de Melo-Diogo D. Correia I. J. Moreira A. F. (2021). Optimization of the gsh-mediated formation of mesoporous silica-coated gold nanoclusters for nir light-triggered photothermal applications. Nanomaterials 11 (8), 1946. 10.3390/nano11081946 34443777 PMC8401642

[B15] Fernandes N. Borges S. Rodrigues C. F. Correia I. J. Moreira A. F. (2025). Polydopamine-coated mesoporous silica/gold nanoclusters for enhanced chemo-photothermal therapy of cancer. Biomater. Adv. 177, 214405. 10.1016/j.bioadv.2025.214405 40644856

[B16] Gaspar V. M. Moreira A. F. Costa E. C. Queiroz J. A. Sousa F. Pichon C. (2015). Gas-generating TPGS-PLGA microspheres loaded with nanoparticles (NIMPS) for co-delivery of minicircle DNA and anti-tumoral drugs. Colloids Surfaces B Biointerfaces 134, 287–294. 10.1016/j.colsurfb.2015.07.004 26209779

[B17] Ghaffarlou M. Rashidzadeh H. Mohammadi A. Mousazadeh N. Barsbay M. Sharafi A. (2024). Photothermal and radiotherapy with alginate-coated gold nanoparticles for breast cancer treatment. Sci. Rep. 14 (1), 13299. 10.1038/s41598-024-60396-w 38858410 PMC11164878

[B18] Gonçalves A. S. (2020). Strategies to improve the photothermal capacity of gold-based nanomedicines. Acta Biomater. 116, 105–137. 10.1016/j.actbio.2020.09.008 32911109

[B19] Gonçalves A. S. Rodrigues C. F. Fernandes N. de Melo-Diogo D. Ferreira P. Moreira A. F. (2022). IR780 loaded gelatin‐PEG coated gold core silica shell nanorods for cancer‐targeted photothermal/photodynamic therapy. Biotechnol. Bioeng. 119 (2), 644–656. 10.1002/bit.27996 34841513

[B20] Gupta N. Malviya R. (2021). Understanding and advancement in gold nanoparticle targeted photothermal therapy of cancer. Biochimica Biophysica Acta (BBA)-Reviews Cancer 1875 (2), 188532. 10.1016/j.bbcan.2021.188532 33667572

[B21] Hao Y. Zhang B. Zheng C. Ji R. Ren X. Guo F. (2015). The tumor-targeting core–shell structured DTX-loaded PLGA@ Au nanoparticles for chemo-photothermal therapy and X-ray imaging. J. Control. Release 220, 545–555. 10.1016/j.jconrel.2015.11.016 26590021

[B22] Hemmer E. Benayas A. Légaré F. Vetrone F. (2016). Exploiting the biological windows: current perspectives on fluorescent bioprobes emitting above 1000 nm. Nanoscale Horizons 1 (3), 168–184. 10.1039/c5nh00073d 32260620

[B23] Hossain M. I. Nanda S. S. Selvan S. T. Yi D. K. (2022). Recent insights into NIR-light-responsive materials for photothermal cell treatments. Nanomaterials 12 (19), 3318. 10.3390/nano12193318 36234446 PMC9565779

[B24] Husni P. Shin Y. Jeon H. Lee E. S. Youn Y. S. Poon C. D. (2023). Development and characterization of pH-responsive nanocarriers for chemo-photothermal combination therapy of acidic tumors. J. Control. Release 359, 52–68. 10.1016/j.jconrel.2023.05.025 37220804

[B25] Iodice C. Cervadoro A. Palange A. Key J. Aryal S. Ramirez M. R. (2016). Enhancing photothermal cancer therapy by clustering gold nanoparticles into spherical polymeric nanoconstructs. Opt. Lasers Eng. 76, 74–81. 10.1016/j.optlaseng.2015.04.017

[B26] Jang W. Yun J. Eyimegwu P. N. Hou J. Byun H. Kim J. H. (2022). Controlling the formation of encapsulated gold nanoparticles for highly reactive catalysts in the homocoupling of phenylboronic acid. Catal. Today 388, 109–116. 10.1016/j.cattod.2020.09.028

[B27] Kadkhoda J. Tarighatnia A. Barar J. Aghanejad A. Davaran S. (2022). Recent advances and trends in nanoparticles based photothermal and photodynamic therapy. Photodiagnosis Photodynamic Therapy 37, 102697. 10.1016/j.pdpdt.2021.102697 34936918

[B28] Kang M. S. Lee S. Y. Kim K. S. Han D. W. (2020). State of the art biocompatible gold nanoparticles for cancer theragnosis. Pharmaceutics 12 (8), 701. 10.3390/pharmaceutics12080701 32722426 PMC7463491

[B29] Kaur J. Singh R. R. Khan E. Kumar A. Joshi A. (2021). Piperine-loaded PLGA nanoparticles as cancer drug carriers. ACS Appl. Nano Mater. 4 (12), 14197–14207. 10.1021/acsanm.1c03664

[B30] Kiri S. Ryba T. (2024). Cancer, metastasis, and the epigenome. Mol. Cancer 23 (1), 154. 10.1186/s12943-024-02069-w 39095874 PMC11295362

[B31] Lee J. Neustrup M. A. Slütter B. O'Mahony C. Bouwstra J. A. van der Maaden K. (2024). Intradermal vaccination with PLGA nanoparticles *via* dissolving microneedles and classical injection needles. Pharm. Res. 41 (2), 305–319. 10.1007/s11095-024-03665-7 38332390 PMC10879229

[B32] Li S.-D. Huang L. (2008). Pharmacokinetics and biodistribution of nanoparticles. Mol. Pharmaceutics 5 (4), 496–504. 10.1021/mp800049w 18611037

[B33] López-Royo T. Sebastián V. Moreno-Martínez L. Uson L. Yus C. Alejo T. (2021). Encapsulation of large-size plasmids in PLGA nanoparticles for gene editing: comparison of three different synthesis methods. Nanomaterials 11 (10), 2723. 10.3390/nano11102723 34685164 PMC8541650

[B34] Mansur H. S. Sadahira C. M. Souza A. N. Mansur A. A. (2008). FTIR spectroscopy characterization of poly (vinyl alcohol) hydrogel with different hydrolysis degree and chemically crosslinked with glutaraldehyde. Mater. Sci. Eng. C 28 (4), 539–548. 10.1016/j.msec.2007.10.088

[B35] Mir F. A. Gani A. Asokan K. (2016). Gamma irradiation studies of composite thin films of poly vinyl alcohol and coumarin. RSC Advances 6 (2), 1554–1561. 10.1039/c5ra15633e

[B36] Moreira A. F. Dias D. R. Costa E. C. Correia I. J. (2017). Thermo-and pH-responsive nano-in-micro particles for combinatorial drug delivery to cancer cells. Eur. J. Pharm. Sci. 104, 42–51. 10.1016/j.ejps.2017.03.033 28347775

[B37] Moreira A. F. Filipe H. A. L. Miguel S. P. Ribeiro M. J. Coutinho P. (2025). Recent advances in smart gold nanoparticles for photothermal therapy. Nanomedicine 20 (11), 1339–1353. 10.1080/17435889.2025.2500912 40329458 PMC12140457

[B38] Nicol J. R. Dixon D. Coulter J. A. (2015). Gold nanoparticle surface functionalization: a necessary requirement in the development of novel nanotherapeutics. Nanomedicine 10 (8), 1315–1326. 10.2217/nnm.14.219 25955125

[B39] Pal S. Koneru J. K. Andreou C. Rakshit T. Rajasekhar V. K. Wlodarczyk M. (2022). DNA-functionalized gold nanorods for perioperative optical imaging and photothermal therapy of triple-negative breast cancer. ACS Appl. Nano Mater. 5 (7), 9159–9169. 10.1021/acsanm.2c01502

[B40] Ramesan M. Sankar S. Kalladi A. J. Labeeba Abdulla A. C. Bahuleyan B. K. (2024). Hydroxyapatite nanoparticles reinforced polyvinyl alcohol/chitosan blend for optical and energy storage applications. Polym. Eng. and Sci. 64 (3), 1378–1390. 10.1002/pen.26623

[B41] Reis E. F. d. Campos F. S. Lage A. P. Leite R. C. Heneine L. G. Vasconcelos W. L. (2006). Synthesis and characterization of poly (vinyl alcohol) hydrogels and hybrids for rMPB70 protein adsorption. Mater. Res. 9, 185–191. 10.1590/s1516-14392006000200014

[B42] Robin B. Albert C. Beladjine M. Legrand F. X. Geiger S. Moine L. (2021). Tuning morphology of Pickering emulsions stabilised by biodegradable PLGA nanoparticles: how PLGA characteristics influence emulsion properties. J. Colloid Interface Sci. 595, 202–211. 10.1016/j.jcis.2021.03.061 33823323

[B43] Rodrigues C. F. Jacinto T. A. Moreira A. F. Costa E. C. Miguel S. P. Correia I. J. (2019a). Functionalization of AuMSS nanorods towards more effective cancer therapies. Nano Res. 12 (4), 719–732. 10.1007/s12274-019-2286-y

[B44] Rodrigues C. F. Reis C. A. Moreira A. F. Ferreira P. Correia I. J. (2019b). Optimization of gold core-mesoporous silica shell functionalization with TPGS and PEI for cancer therapy. Microporous Mesoporous Mater. 285, 1–12. 10.1016/j.micromeso.2019.04.064

[B45] Salimi M. Mosca S. Gardner B. Palombo F. Matousek P. Stone N. (2022). Nanoparticle-mediated photothermal therapy limitation in clinical applications regarding pain management. Nanomaterials 12 (6), 922. 10.3390/nano12060922 35335735 PMC8951621

[B46] Schrand A. M. Rahman M. F. Hussain S. M. Schlager J. J. Smith D. A. Syed A. F. (2010). Metal‐based nanoparticles and their toxicity assessment. Wiley Interdisciplinary Reviews Nanomedicine Nanobiotechnology 2 (5), 544–568. 10.1002/wnan.103 20681021

[B47] Sharma S. (2016). PLGA-based nanoparticles: a new paradigm in biomedical applications. TrAC Trends Analytical Chemistry 80, 30–40. 10.1016/j.trac.2015.06.014

[B48] Silva A. T. C. R. (2015). Synthesis, characterization, and study of PLGA copolymer *in vitro* degradation. J. Biomaterials Nanobiotechnology 6 (1), 8–19. 10.4236/jbnb.2015.61002

[B49] Soppimath K. S. Aminabhavi T. M. Kulkarni A. R. Rudzinski W. E. (2001). Biodegradable polymeric nanoparticles as drug delivery devices. J. Controlled Release 70 (1-2), 1–20. 10.1016/s0168-3659(00)00339-4 11166403

[B50] Swider E. Koshkina O. Tel J. Cruz L. J. de Vries I. J. M. Srinivas M. (2018). Customizing poly (lactic-co-glycolic acid) particles for biomedical applications. Acta Biomater. 73, 38–51. 10.1016/j.actbio.2018.04.006 29653217

[B51] Tabesh F. Haghverdi G. Devarakonda K. P. Massoud T. F. Paulmurugan R. (2024). Synthesis, characterization, and application of a biocompatible gene delivery nanocarrier constructed from gold nanostars and a chitosan–cyclodextrin–poly (ethylene imine) graft polymer. Mater. Adv. 5 (20), 8007–8016. 10.1039/d4ma00433g

[B52] Tatykhanova G. S. Tuleyeva R. N. Nurakhmetova Z. A. Gizatullina N. N. Krasnoshtanov V. K. Kaldybekov D. B. (2025). Polymer‐Protected gold nanoparticles for photothermal treatment of ehrlich adenocarcinoma: *in vitro* and *in vivo* studies. Macromol. Chem. Phys. 226 (4), 2400128. 10.1002/macp.202400128

[B53] Tonbul H. Çapan Y. (2023). Hybrid PLGA nanoparticles as advanced drug delivery and theranostic applications, in poly (lactic-co-glycolic acid)(PLGA) nanoparticles for Drug Delivery. Elsevier, 417–431.

[B54] Vazirieh Lenjani S. Mayer M. Wang R. Dong Y. Fery A. Sommer J. U. (2022). Importance of electrostatic forces in supracolloidal self-assembly of polymer-functionalized gold nanorods. J. Phys. Chem. C 126 (32), 14017–14025. 10.1021/acs.jpcc.2c04930

[B55] Wang H. Zhao P. Su W. Wang S. Liao Z. Niu R. (2010). PLGA/polymeric liposome for targeted drug and gene co-delivery. Biomaterials 31 (33), 8741–8748. 10.1016/j.biomaterials.2010.07.082 20727587

[B56] Wang J. Helder L. Shao J. Jansen J. A. Yang M. Yang F. (2019). Encapsulation and release of doxycycline from electrospray-generated PLGA microspheres: effect of polymer end groups. Int. Journal Pharmaceutics 564, 1–9. 10.1016/j.ijpharm.2019.04.023 30978487

[B57] Xi J. Wang W. Da L. Zhang J. Fan L. Gao L. (2018). Au-PLGA hybrid nanoparticles with catalase-mimicking and near-infrared photothermal activities for photoacoustic imaging-guided cancer therapy. ACS Biomaterials Sci. and Eng. 4 (3), 1083–1091. 10.1021/acsbiomaterials.7b00901 33418792

[B58] Yang W. Xia B. Wang L. Ma S. Liang H. Wang D. (2021). Shape effects of gold nanoparticles in photothermal cancer therapy. Mater. Today Sustain. 13, 100078. 10.1016/j.mtsust.2021.100078

[B59] Yang N. Kang Y. Cong Y. Wang X. Yao C. Wang S. (2023). Controllable gold nanocluster–emulsion interface for direct cell penetration and photothermal killing. Adv. Mater. 35 (50), 2208349. 10.1002/adma.202208349 36271742

[B60] Yao X. Teng R. Jin M. Wan D. (2023). Dendritic molecular template mediates and directs self-assembly of uniform gold nanoclusters to one-pot afford 3D-Supported active catalysts. ACS Appl. Polym. Mater. 5 (9), 6842–6850. 10.1021/acsapm.3c00842

[B61] Zhang W. Mehta A. Tong Z. Esser L. Voelcker N. H. (2021). Development of polymeric nanoparticles for blood–brain barrier transfer—strategies and challenges. Adv. Sci. 8 (10), 2003937. 10.1002/advs.202003937 34026447 PMC8132167

[B62] Zhang H. Tang X. Jiang Z. (2025). Next-generation Nanomaterial-based phototherapeutic strategies for tumor: from NIR responsiveness to smart activation. Nanomedicine 20, 1–25. 10.1080/17435889.2025.2532357 40662637 PMC12320853

[B63] Zhi K. Raji B. Nookala A. R. Khan M. M. Nguyen X. H. Sakshi S. (2021). PLGA nanoparticle-based formulations to cross the blood–brain barrier for drug delivery: from R&D to cGMP. Pharmaceutics 13 (4), 500. 10.3390/pharmaceutics13040500 33917577 PMC8067506

[B64] Zhuo Y. (2022). Gold nanocluster and indocyanine green based triple-effective therapy for MRSA infected central nervous system. Appl. Mater. Today 27, 101453. 10.1016/j.apmt.2022.101453

