# Supplementary material for: Development of gold nanoparticles stabilized by PLGA and PVA for application in photothermal therapy
Source: Front Cell Dev Biol. 2026 Feb 5;14:1759401. doi: 10.3389/fcell.2026.1759401 (PMC12916575; doi:10.3389/fcell.2026.1759401)
Supplement: Supplementary file 1 [file DataSheet1.docx]

**Development of gold nanoparticles stabilized by PLGA and PVA for application in photothermal therapy.**

Soraia Borges^1^, Natanael Fernandes^1,2^, and André F. Moreira^1,3,*^

^1^ RISE-Health, Departamento de Ciências Médicas, Faculdade de Ciências da Saúde, Universidade da Beira Interior, Covilhã, Portugal

^2^ AEROG-LAETA, Aerospace Sciences Department, Universidade da Beira Interior, Covilhã, Portugal

^3^ BRIDGES - Biotechnology Research, Innovation, and Design of Health Products, Polytechnic of Guarda, Av. Dr. Francisco Sá Carneiro, 50, 6300-559 Guarda, Portugal

* Corresponding author: e-mail: [afmoreira@ipg.pt](mailto:afmoreira@ipg.pt)


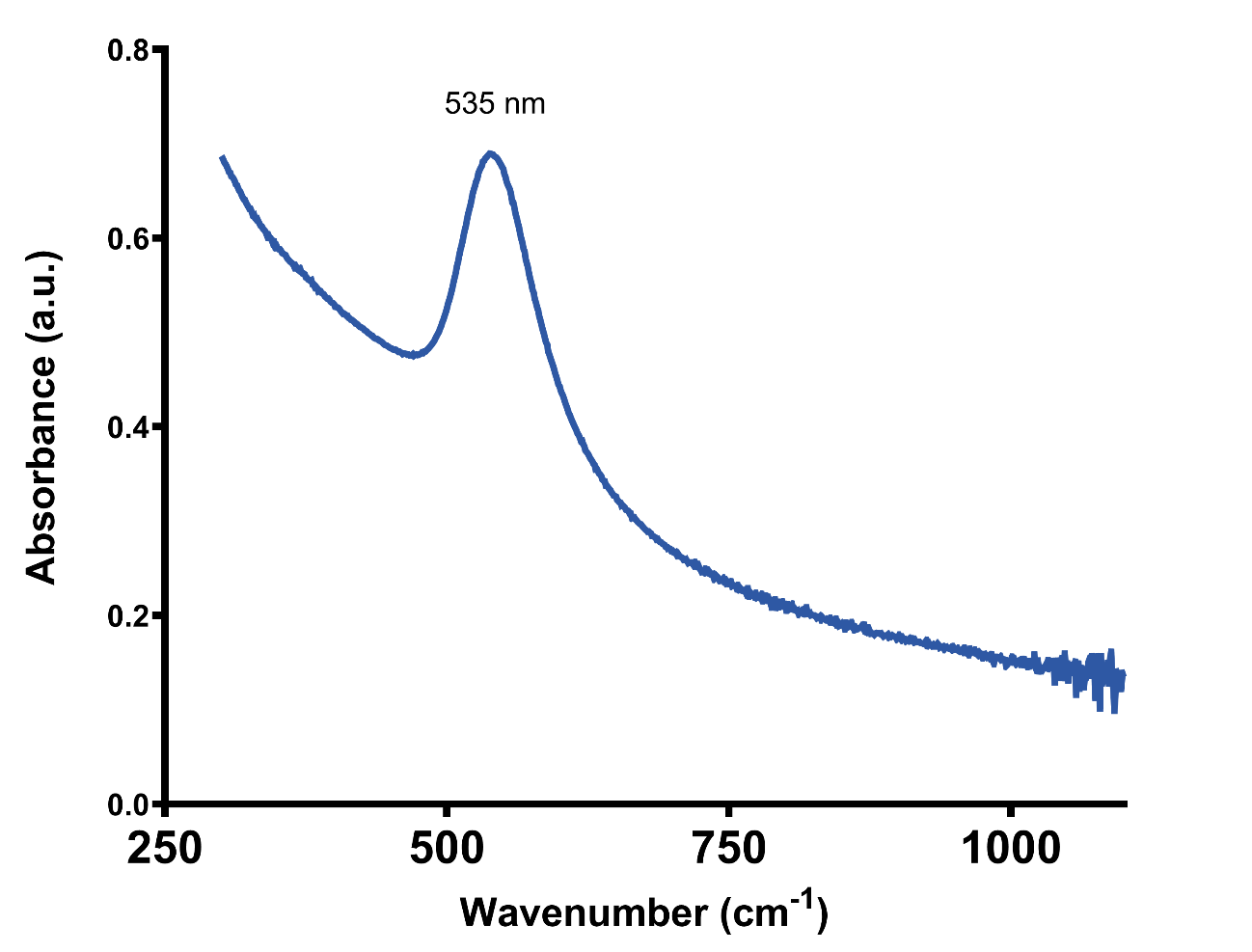


Figure S1 – UV-vis spectrum of spherical gold nanoparticles showing the characteristic absorption peak at 535 nm.


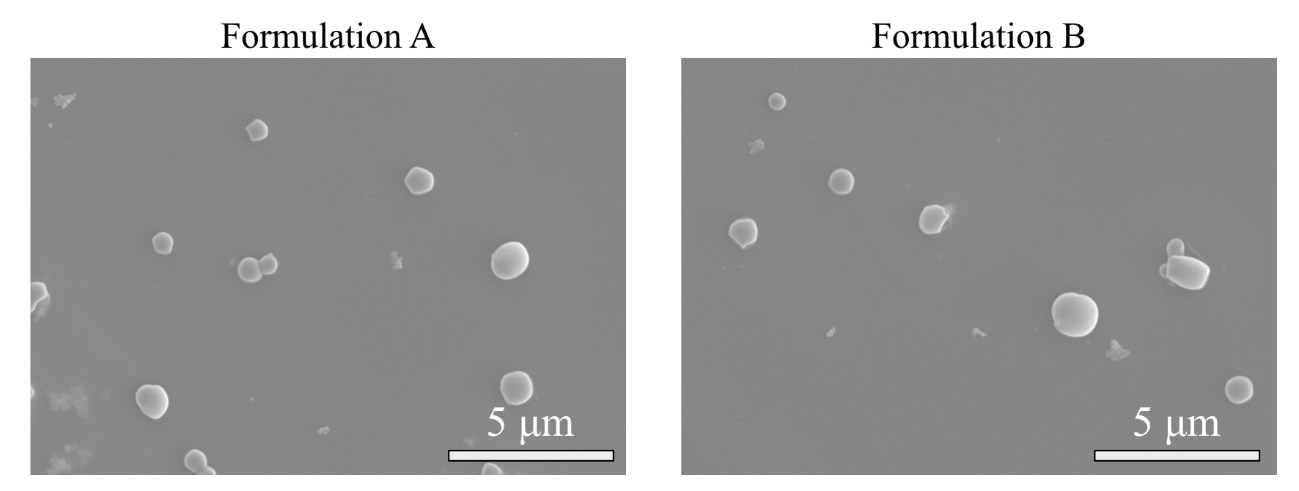


Figure S2 – SEM images of Formulation A and B before irradiation with the NIR laser.
